# Supplementary material for: Natural Thiols, but Not Thioethers, Attenuate Patulin-Induced Endoplasmic Reticulum Stress in HepG2 Cells
Source: Toxins (Basel). 2021 Oct 14;13(10):727. doi: 10.3390/toxins13100727 (PMC8537938; doi:10.3390/toxins13100727)
Supplement: Supplementary file 1 [file toxins-13-00727-s001.zip › toxins-1355390-supplementary.pdf]

Natural Thiols, But Not Thioethers, Attenuate Patulin-Induced Endoplasmic Reticulum Stress in HepG2 Cells

Hye Mi Kim, Hwa Young Choi, Gun Hee Cho, Ju Hee Im, Eun Young Hong and Hyang Sook Chun*

Table S1. Primers used in reverse transcription-quantitative real-time polymerase chain reactions

Gene	Primer Sequence	Product Size (Base Pairs)
BIP (GRP78)	5′-GGTGACCTGGTACTGCTTGATG-3′<br>5′-CCTTGGATTCAGTTTGGTCATG-3′	84
CHOP (GADD153)	5′-CTTGGCTGACTGAGGAGGAG-3′<br>5′-TCACCATTCGGTCAATCAGA-3′	312
XBP1	5′-CCTTGGATTCAGTTTGGTCATG-3′<br>5′-GGGGCTTGGTATATATGTGG-3′	442<br>416
*usXBP1**	5′-CAGCACTCAGACTACGTGCA-3′<br>5′-ATCCATGGGGAGATGTTCTGG-3′	76
*sXBP1***	5′-CTGAGTCCGAATCAGGTGCAG-3′<br>5′-ATCCATGGGGAGATGTTCTGG-3′	59
ACTB	5′-TCATCACCATTGGCAATGAG-3′<br>5′-CACTGTGTTGGCGTACAGGT-3′	154

*us*XBP1* (unspliced *XBP1*); **s*XBP1* (spliced *XBP1*)
